# Electrolysis for the Destruction of Hairs and Moles

**Published:** 1892-01

**Authors:** M. B. Hutchins

**Affiliations:** Lecturer on Dermatology, Atlanta Medical College, Atlanta, Ga.


### ELECTROLYSIS FOR THE DESTRUCTION OF HAIRS AND MOLES.

### CASES.

By M. B. HUTCHINS, M. D.,
Lecturer on Dermatology, Atlanta Medical College,
Atlanta, Ga.

About ten years ago Dr. Michel, of St. Louis, reported successful results of electrolysis in the treatment of trichiasis. Dr. Hardaway, of the same city, deserves the credit of applying the same measures to the treatment of growths of superfluous hairs, as met with in the practice of dermatology. The following case from my practice may prove of interest :

### HYPERTRICHOSIS.

Mrs. ———, age 43, consulted me on October 30, 1890, with condition to be described:

Several large, dark moles on lower half of face. Growing in these, numerous long hairs, from almost colorless to deep black in shade. On each angle of chin others were present, quite abundantly, and there were also many finer and paler hairs over the chin. Quite a decided growth on upper lip, especially near the corners of the mouth, where they were coarse. Those in the moles and on the chin were more or less curled or twisted from the patient's efforts to make them "lie down." She thought increased growth resulted from pulling the hairs out.

History of keloid on chest from caustic application. This was excised eighteen months previous, and the site of the majority of the stitches was marked by hypertrophic scar tissue. This tendency to so-called "false keloid" warned me that the use of the electrolytic needle would have to be very cautious, otherwise we might get a lot of these growths at the points operated upon.

The first operation was done December 12th, about six weeks later. Having no miliamperemeter, it was necessary to estimate the current of electricity by "cells" alone. A Kidder battery was the first used, five cells furnishing sufficient current. To the positive pole was attached an ordinary sponge electrode ; to the negative a pencil-shaped needle holder containing a fine sewing needle—which answers every purpose. Upon this holder was a spring, which had to be pressed down to complete the circuit through the needle. At first this spring was bound down and the circuit completed or broken simply by letting the patient take hold of or let go the sponge electrode. Later it was found best to let the spring regulate the circuit, the patient only letting go the sponge in order to wet it. (The *negative* pole is the one to which to attach the needle, because, if it were attached to the *positive*, a black point, as will be seen later, would result from the chemical action taking place.)

The sponge electrode was kept well wet with water from a basin near. Before completing the circuit the needle was gently inserted by the side of and in the direction of the hair (they do not grow straight out—perpendicular to the skin) until a faint resistance, the "bottom" of the follicle, was felt. Completion of the circuit was followed by a little frothing around the point of the needle, this being the more marked where the needle had penetrated exactly to the "bottom" of the follicle. After fifteen seconds to probaby a minute the circuit was broken and traction made upon the hair with forceps. If it came out easily, we could be sure the papilla was destroyed and that another hair would not grow. If the hair proved hard to extract, the needle was re-inserted and the current again applied. It was noticed that there was less pain when the needle was properly inserted, but at best it is rather suggestive, at first, of a bee sting. The pain, however, is only at the instant of completion of the circuit, and there follows a somewhat benumbing secondary effect. The punctured skin became red and tended to form mosquito-bite-like lesions. For the purpose of relieving the irritation, the following was applied at intervals, both during and after the operations:

℞. Zinci oxidi pulv., ʒi.

Ether. sulph., ʒii.

Aquæ, ʒvi.

M. Sig: Shake and apply.

On December 12th ten hairs were removed, and twelve the next day. The patient returned to her home, out of the city, and the next *seance* was not until February 23, 1891. (It is said that ten per cent. of the hairs will return, but I think our percentage was much lower than that.) This time I used a borrowed battery, and made the mistake of attaching the needle to the *positive* pole, thus producing *a black point*. The needle was immediately changed to the *negative* pole. Six cells first used, then eight. On February 23d, removed twelve hairs ; on the 24th, sixteen, using only six cells ; on 25th, fifteen removed, and the large mole on right side of chin was transfixed with the needle twice, the punctures at right angles to each other, six cells. Some hairs in the moles were directly operated upon, while others were left, to see if the operation on the moles would cause the hairs to be destroyed. It was found, later, that some *were* thus destroyed. During the passage of the current through the moles, they changed from dark brown to yellowish white. On February 27th five hairs were removed, using six Law cells, sal ammoniac charge.

Next *seance* March 21. Twenty-one hairs removed, six Law cells. Moles not much smaller. Black point treated with needle from negative pole to see if a reversed chemical action would cause its disappearance. A lotion of oxide of zinc, subnitrate of bismuth, alcohol and water was now being used for the soothing effect. March 23d, twenty hairs from chin. No large, black ones remained. On 24th, ten small hairs from chin, and five, large, from each " side " of upper lip. Mole right side again treated. On 25th, five each side of lip. Black point again treated. On May 13th, 14th and 15th other operations ; larger hairs on lip, smaller on chin. Moles disappearing, but patient had rather given up the idea of having them further treated. On September 7th and 8th the work was completed, only a few fine, lanugo hairs remaining on lip and chin. From the removal of ten at the first sitting, the patient gradually reached the ability to stand from forty to fifty being removed at a time. The re-growths which took place during the intervals amounted practically to *nothing*. At last operation the black point had entirely disappeared.

When one undertakes to count hairs he is going to be very much surprised at how far the number exceeds his estimate. I found it so in this case. There did not seem to be *many*. From the chin one hundred and eighty-five (185) were removed. From the upper lip one hundred and sixty-seven (167). From face, neck and "scattering," twenty-eight (28). Total 380.

Sufficient time has elapsed to justify the belief that the operation will prove a complete success.

### MOLES.

The effect of only a few applications of the current to the moles in the above case proved that they could have thus been destroyed. Three cases were treated by electrolysis, but two of them did not continue the sittings, only attending once for treatment.

Case 1.—Mr. ——, age 21. Just above and to the right of the upper lip a round, pinkish, solid growth the size of an English pea. Present all his life. The mole was pierced nearly through its base, transversely, with a large spear-pointed copper needle and a current of ten cells used. Then the needle was reinserted at a right angle, transversely, to first puncture and the same current employed; current each time allowed to pass for about thirty seconds. Some blanching of the mole and considerable frothing along the course of the needle were produced by the action of the current. Pain was only moderate and temporary.

A week later the growth seemed a little smaller, and sensation was much blunted. The current from eight cells now used through a small sewing needle. Frothing around needle as at previous sitting. Twelve days later all that remained of the growth were two small pin-head sized dark elevations. There was no further treatment, as they gave promise of gradual disappearance of themselves.

Two other cases were similarly treated, but the result is not known. The growth in one of these cases had partially declined after the use of electrolysis a year previous.

1½ *Edgewood Avenue.*

